# *Clostridioides difficile* infection in rifampin-sensitive pulmonary tuberculosis: a retrospective comparative study

**DOI:** 10.3389/fcimb.2026.1926121

**Published:** 2026-10-28

**Authors:** Yongheng Liu, Cuizhu Ren, Yong Guo, Adili Jumahan, Tuersun Yueriyeti, Jianzhong Yang, Chuanjian Shi

**Affiliations:** 1 State Key Laboratory of Pathogenesis, Prevention and Treatment of High Incidence Diseases in Central Asia, Hami, Xinjiang, China; 2 Clinical Laboratory, Hami Central Hospital, Hami, Xinjiang, China; 3 Infectious Disease Department, Hami Central Hospital, Hami, Xinjiang, China

**Keywords:** case series, clostridioides difficile infection, diarrhea, proton pump inhibitors, pulmonary tuberculosis, rifampin-sensitive tuberculosis

## Abstract

**Background:**

*Clostridioides difficile infection* (CDI) is an important cause of hospital-acquired diarrhea but remains poorly characterized during tuberculosis treatment. Patients with pulmonary tuberculosis may be particularly vulnerable because of prolonged treatment, hospitalization, comorbidities, proton pump inhibitor (PPI) exposure, and treatment-related disruption of the intestinal microbiota.

**Objective:**

This study aimed to characterize CDI in patients with rifampin-sensitive pulmonary tuberculosis and explore clinical factors associated with its occurrence.

**Methods:**

This retrospective comparative study included 30 adults hospitalized with rifampin-sensitive pulmonary tuberculosis between June 2024 and January 2025 at a tertiary infectious disease hospital in western China. Ten patients with clinically diagnosed, PCR-positive CDI were compared with 20 contemporaneous tuberculosis patients without CDI. Demographic characteristics, clinical manifestations, laboratory parameters, medication exposure, and in-hospital outcomes were extracted from medical records. Group comparisons and exploratory multivariable logistic regression were performed to identify factors associated with CDI.

**Results:**

Patients with CDI had significantly lower body mass index (P = 0.009), mean corpuscular volume (P = 0.014), and serum albumin levels (P = 0.041) than those without CDI. Other hematological, inflammatory, hepatic, and renal parameters were generally comparable between groups. None of the three significant variables remained independently associated with CDI in multivariable analysis. PPI exposure was common among patients with CDI, with nine of ten having received PPIs for at least two weeks during anti-tuberculosis treatment. Seven patients with CDI improved during hospitalization, two were transferred to higher-level hospitals, and one died.

**Conclusion:**

CDI represents an important diagnostic consideration in patients with rifampin-sensitive pulmonary tuberculosis who develop gastrointestinal symptoms during treatment. Lower BMI, MCV, and serum albumin characterized patients with CDI but were not independently associated with infection after adjustment. These findings highlight the importance of clinical vigilance and timely CDI testing in this population.

## Introduction

Tuberculosis (TB) remains a major global health threat, with over 10 million new cases annually and high mortality in low- and middle-income countries (Bagcchi, 2023). Meanwhile, *Clostridioides difficile* infection (CDI) has become the most common hospital-acquired gastrointestinal infection, particularly in the elderly and hospitalized populations (Miller et al., 2011; Bilgin et al., 2020). Although both diseases are prevalent among vulnerable individuals, their coexistence is rarely reported. The nonspecific symptoms of CDI—such as diarrhea and anorexia—often overlap with TB-related gastrointestinal complaints, which may delay recognition (Bessaleli and Scheinfeld, 2018).

Known risk factors for CDI include advanced age, recent hospitalization, broad-spectrum antibiotic exposure, and proton pump inhibitor (PPI) use (Pham and Hua, 2021). PPI use has been associated with an increased risk of CDI, although the strength and causality of this association remain debated (Finke et al., 2025)among. Patients with rifampin-sensitive pulmonary tuberculosis may experience several overlapping CDI-associated exposures, including older age and comorbidities, prolonged hospitalization, PPI use for gastroprotection, non-tuberculous antibiotics for secondary infections, and prolonged multidrug anti-tuberculosis therapy (Hu et al., 2019; Finke et al., 2025). Previous studies have shown that conventional anti-tuberculosis treatment can induce marked and persistent alterations in intestinal microbial composition, and rifampin appears to be an important contributor to these treatment-related microbiota changes. These overlapping exposures provide a biologically plausible basis for dysbiosis while making it difficult to attribute CDI to any single medication (Wipperman et al., 2017; Hu et al., 2019). However, CDI during treatment of rifampin-sensitive tuberculosis remains poorly characterized.

Despite the increasing awareness of CDI in hospitalized populations, its occurrence in patients undergoing anti-tuberculosis therapy remains poorly characterized. Most existing data are limited to individual case reports, often involving diagnostic uncertainty or misattribution of symptoms to tuberculosis or drug-related effects. For example, a patient with tuberculous pericarditis developed CDI following rifampin exposure, but diarrhea was initially interpreted as a side effect of antituberculous drugs (Sun et al., 2013). Similarly, CDI presenting as appendicitis in an immunocompromised host highlights the diverse and often misleading clinical manifestations of this infection (Pham and Hua, 2021). In tuberculosis cohorts, gastrointestinal symptoms such as diarrhea, anorexia, and abdominal discomfort are frequently considered nonspecific, contributing to underdiagnosis. Moreover, confirmatory stool testing is not routinely pursued unless clinicians maintain a high level of suspicion, delaying appropriate management (Ahlquist, 2019). To date, no retrospective study has systematically described the clinical profile, diagnostic process, and treatment outcomes of clinically classified, PCR-positive CDI in patients with rifampin-sensitive tuberculosis.

To address the lack of structured data, we conducted a retrospective analysis of ten inpatients with rifampin-sensitive pulmonary tuberculosis who were clinically classified as having PCR-positive *Clostridioides difficile* infection. This study is, to our knowledge, the first to integrate microbiological diagnosis, detailed PPI exposure history, imaging review, and outcome classification in this setting. By combining clinical, pharmacological, and radiologic data from a TB-specialized hospital, we aimed to improve recognition of this rare but underappreciated overlap.

## Methods

### Case selection and data source

This retrospective comparative study included 30 adults hospitalized with rifampin-sensitive pulmonary tuberculosis at a tertiary infectious disease hospital in western China between June 2024 and January 2025. Ten patients were classified into the CDI group, and 20 contemporaneous patients from the same period served as non-CDI controls. Controls were not individually matched for age, sex, or length of stay. The same eligibility criteria were applied retrospectively to both groups.

Pulmonary tuberculosis was diagnosed based on clinical findings, chest imaging, acid-fast staining, nucleic acid amplification testing, and mycobacterial culture. CDI classification required a clinical diagnosis supported by stool PCR detection of toxigenic C. difficile. Patients whose gastrointestinal symptoms were clearly attributable to another established infectious or noninfectious cause were excluded. Because PCR cannot distinguish colonization from active toxin-mediated infection, possible colonization could not be completely excluded. Non-CDI controls had no clinical evidence or positive test for CDI during hospitalization.

Demographic, clinical, treatment, imaging, microbiological, and medication-exposure data were extracted from electronic medical records. Medication and timing information, including rifampin, non-tuberculous antibiotics, PPI exposure, gastrointestinal symptoms, stool testing, and hospital outcomes, was recorded when clearly documented in the source records.

### Data collection and definitions

Data collected included age, sex, comorbidities, tuberculosis classification, bacteriological findings, anti-tuberculosis regimens, PPI exposure, non-tuberculous systemic antibacterial exposure, gastrointestinal symptoms, stool PCR results, CDI management, and in-hospital outcomes. PPI exposure was defined as continuous use for more than three days before CDI onset or diagnosis. Non-tuberculous antibacterial exposure was recorded when documented in the medical records; drug class, indication, dose, duration, and timing were extracted when available.

CDI-group classification required a clinician-recorded diagnosis supported by stool PCR detection of toxigenic *C. difficile*. Diarrhea (McDonald et al., 2018), when documented, was defined as at least three unformed stools within 24 hours. Diagnostic delay was defined as the interval between documented diarrhea onset and stool PCR testing. Non-CDI controls had no clinical or laboratory evidence of CDI during the index hospitalization.

Eligibility criteria for both groups were age ≥18 years, rifampin-sensitive pulmonary tuberculosis, and hospitalization during the study period. Patients with incomplete clinical records or gastrointestinal symptoms clearly attributable to another established cause, including laxative use, inflammatory bowel disease, gastrointestinal malignancy, or a documented enteric infection, were excluded. Extraintestinal infections were not exclusion criteria because they could coexist with CDI and were considered potential sources of systemic inflammation and non-tuberculous antimicrobial exposure. Outcomes were assessed until discharge, transfer, or in-hospital death.

### Ethics and statistical analysis

This study was approved by the institutional ethics committee of the participating hospital. As a retrospective analysis of anonymized clinical data, the requirement for informed consent was waived.

Descriptive statistics were used to summarize demographic features and clinical characteristics. Comparative statistical analyses were performed between the CDI and non-CDI groups. Categorical variables were presented as frequencies and percentages and compared using the χ² test or Fisher’s exact test, as appropriate. Continuous variables were assessed for normality; normally distributed data were expressed as mean ± standard deviation and compared using the independent-samples t test, whereas non-normally distributed data were expressed as median (interquartile range) and compared using the Mann–Whitney U test.

Variables with P < 0.05 in univariate analyses were entered into a multivariable binary logistic regression model to explore factors potentially associated with CDI. Statistical significance was set at a two-sided P value < 0.05.

All data analyses were performed using IBM SPSS Statistics version 26.0 (IBM Corp., Armonk, NY, USA). This research involved a retrospective review of medical records and imaging data from hospitalized patients and did not involve any direct interventions, treatment modifications, or patient enrollment.

## Results

### Patient characteristics

Ten patients with rifampin-sensitive pulmonary tuberculosis were assigned to the clinically classified, PCR-positive CDI group. The median age was 74.5 years (range, 48–89 years), and 7 patients (70%) were male. All patients were of Chinese ethnicity, including 8 Han, 1 Uyghur, and 1 Hui. Tuberculosis was bilateral in 7 cases and involved the right lung in 3 cases. Six patients had complications such as bronchial tuberculosis, pleuritis, or cavitary lesions. Comorbidities were common: 8 patients had chronic respiratory diseases (e.g., chronic obstructive pulmonary disease or pulmonary heart disease), and 6 had nutritional or metabolic disorders (e.g., hypoproteinemia, malnutrition, or electrolyte imbalance). Hypertension (n=4), hepatitis B (n=3), and gastrointestinal conditions (e.g., gastroesophageal reflux, chronic gastritis) were also frequently observed. One patient had schizophrenia, and one had a recent intracerebral hemorrhage. All patients were immunocompetent, and none had HIV coinfection. Details in **Table 1**.

**Table 1:** Comparison of baseline characteristics between CDI and non-CDI groups.

Characteristics	CDI (n = 10)	Non-CDI (n = 20)	Statistic	*P* value
Sex, n (%)			χ² = 2.400	0.121
Male	7 (23.33)	8 (26.67)		
Female	3 (10.00)	12 (40.00)		
Age (years), mean ± SD	69.80 ± 11.45	72.10 ± 10.67	*t* = −0.756	0.456
BMI (kg/m²), mean ± SD	19.80 ± 3.01	23.70 ± 3.71	*t* = −2.817	0.009
Length of hospital stay (days), mean ± SD	28.80 ± 20.02	20.60 ± 11.24	*t* = −1.927	0.064
Comorbidities, n (%)			χ² = 6.277	0.164
Hypertension	2 (6.67)	1 (3.33)		
Cardiovascular disease	2 (6.67)	6 (20.00)		
COPD	3 (10.00)	4 (13.33)		
Malnutrition / hypoalbuminemia	6 (20.00)	4 (13.33)		
Other	3 (10.00)	13 (43.33)		
Type of pulmonary tuberculosis, n (%)			χ² = 0.036	0.849
Initial episode	8 (26.67)	18 (60.00)		
Recurrent	2 (6.67)	2 (6.67)		

### Respiratory manifestations and pulmonary imaging

All ten patients exhibited classical respiratory symptoms of pulmonary tuberculosis. Cough (n=9) and sputum production (n=8) were the most common complaints. Dyspnea or shortness of breath occurred in 6 patients, while fatigue, chest tightness, and reduced appetite were also frequently reported. Two patients (Cases 5 and 10) presented with hemoptysis, and three (Cases 4, 6, and 10) experienced low-grade fever.

Chest CT was performed in all cases. Bronchiectasis was observed in 6 patients (Cases 2, 4, 5, 6, 9, and 10), often associated with adjacent patchy opacities or consolidation. Cavitary lesions were present in 3 patients (Cases 2, 3, and 9), among whom Case 9 exhibited a new cavity during follow-up. Nodular lesions were seen in 4 cases, and miliary nodules were noted in Case 8. Interstitial fibrotic changes with honeycombing appeared in Case 9, along with emphysematous changes. Patchy or flake-like infiltrates were seen in 5 patients. Bronchoscopic examination or percutaneous lung biopsy was performed in 3 cases (Cases 2, 3, and 10), all confirming granulomatous inflammation consistent with active tuberculosis(**Figure 1**). Radiological features are further summarized in **Table 2**.

**Figure 1:**
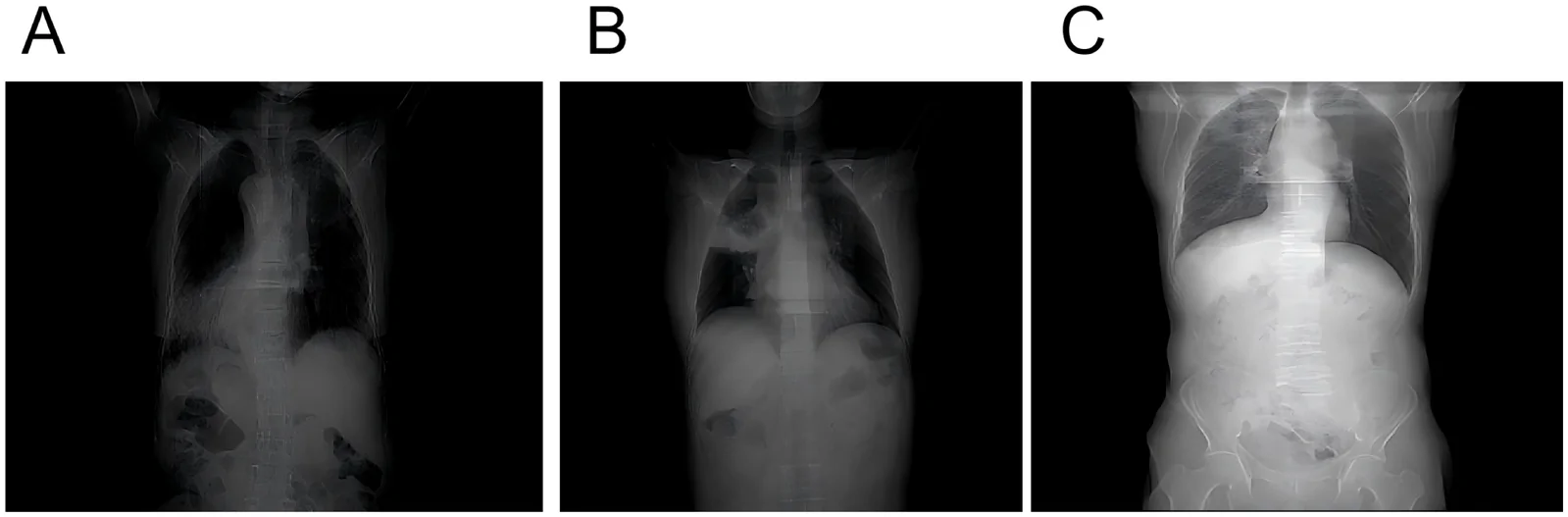
displays representative CT images of three selected cases: **(A)** a newly formed cavity in the right upper lobe with bronchiectasis and nodular lesions (Case 9, 10/23/2024); **(B)** bilateral bronchiectasis with peribronchial infiltrates and cavitation (Case 2, 06/20/2024); and **(C)** miliary nodules and right-sided pleural thickening with effusion (Case 8, 09/01/2024).

**Table 2:** Summary of case characteristics.

Case no.	Sex/Age	Key symptoms & duration	Medical history	Chest CT	MTB PCR	Rifampin resistance	Bronchoscopy/Biopsy	Initial diagnosis
1	Male/89	Cough, sputum (onset ~early June)	Hypertension, COPD, gut flora imbalance	Bilateral infiltrates	Positive	No	No	Pulmonary TB, both lungs
2	Female/58	Cough, sputum (onset ~late June)	Hypertension, HBV (recovered), hypoxemia, dysbiosis	Right lung nodules, cavities	Positive	No	Yes	Pulmonary TB, right lung with bronchial TB and cavities
3	Male/68	Dry cough, dyspnea (onset ~early July)	BPH, gut flora imbalance	Right upper lobe cavity	Positive	No	Yes	Recurrent TB, right upper lobe, cavity
4	Male/74	Cough, dyspnea, weight loss, diarrhea (chronic)	COPD, heart disease, fungus, ICH, dysbiosis	Patchy infiltrates, bronchiectasis	Positive	No	No	Pulmonary TB, extensive with complications
5	Male/48	Cough, weight loss, diarrhea (chronic)	Malnutrition, anemia, infection, schizophrenia	Patchy infiltrates, bronchiectasis	Positive	No	No	Pulmonary TB, both lungs
6	Male/74	Cough, sputum, diarrhea (1+ week)	COPD, CHF, dysbiosis, GI disorders	Bilateral opacities, bronchiectasis	Positive	No	Yes	Pulmonary TB, with pleuritis and bronchial TB
7	Male/63	Cough, diarrhea (within 1 week)	Hypertension, hyperuricemia, HBV	Mild bilateral changes	Positive	No	Yes	Pulmonary TB, initial diagnosis
8	Male/75	Fatigue, diarrhea (~3 weeks)	COPD, hepatic dysfunction, BPH	Cavity and pleuritis (right upper lobe)	Positive	No	No	Pulmonary TB, right upper with pleuritis
9	Female/79	Cough, dyspnea, diarrhea (>2 weeks)	Malnutrition, liver dysfunction, dysbiosis	Patchy bilateral changes	Positive	No	No	Pulmonary TB, both lungs
10	Female/70	Cough, dyspnea, hemoptysis, diarrhea (chronic)	GERD, gastritis, malnutrition, anemia	Bilateral consolidation, cavity	Positive	No	Yes	Pulmonary TB with bronchial TB

### Digestive symptoms and CDI-related features

Among the ten patients included, six (60%) developed new-onset diarrhea during hospitalization, while abdominal distension and low-grade fever were reported in five patients each, and abdominal pain in four. According to stool microscopy and smear results, leukocytosis was observed in eight patients, fecal occult blood tested positive in six, and mucous stools were recorded in nine. All patients fulfilled the diagnostic criteria for CDI, defined as the presence of ≥3 unformed stools within 24 hours combined with a positive stool PCR result for *C. difficile* toxin genes.

Despite this, clinical suspicion of CDI before laboratory confirmation remained low. Abdominal CT findings in several patients revealed nonspecific intestinal wall thickening or colonic dilatation, supporting inflammatory involvement. A summary of digestive symptoms, stool test results, and diagnostic timelines is provided in **Table 3**.

**Table 3:** Laboratory findings, CDI confirmation and digestive symptoms.

Case no.	CRP (mg/L)	Alb (g/L)	WBC (×10^9^/L)	Occult Blood (+)	Abdominal CT findings	Diarrhea	Distension	Pain	Fever	C. difficile PCR	Outcome
1	10.0 ↑	36.8 ↓	5.28	–	–	No	No	No	No	Positive	Recovered
2	1.22	42.9	4.97	–	–	No	No	No	No	Positive	Residual symptoms
3	11.83 ↑	37.8 ↓	6.94	–	–	No	No	No	No	Positive	Transferred
4	52.05 ↑	33.20 ↓	10.86↑	Yes	Colonic wall thickening	Yes	Yes	Yes	Yes	Positive	Death
5	106.72 ↑	24.00 ↓	27.8 ↑	Yes	–	Yes	Yes	No	No	Positive	Residual symptoms
6	136.75 ↑	23.90 ↓	15.31 ↑	–	Mild distension	Yes	Yes	Yes	Yes	Positive	Transferred
7	3.26	37.70↓	3.91	Yes	–	Yes	Yes	No	No	Positive	Improved
8	113.73 ↑	36.70↓	5.17	Yes	Bowel gas-fluid level	Yes	Yes	No	No	Positive	Transferred
9	129.78↑	61.70	10.82 ↑	Yes	Colonic dilatation	Yes	Yes	Yes	Yes	Positive	Improved
10	45.47↑	26.70	5.54	Yes	Segmental bowel inflammation	Yes	Yes	Yes	Yes	Positive	Transferred

↑ indicates a value above the local laboratory reference range; ↓ indicates a value below the local laboratory reference range.

### Hematological and inflammatory parameters

Comparisons of hematological and inflammatory parameters between the CDI and non-CDI groups are presented in **Table 4**. Overall, most routine blood cell counts and inflammatory markers did not differ significantly between the two groups.

**Table 4:** Comparison of hematological and inflammatory parameters between CDI and non-CDI groups.

Parameters	CDI (n = 10)	Non-CDI (n = 20)	Statistic	*P* value
White blood cell count (×10^9^/L)	6.24 (5.12, 14.00)	5.98 (5.21, 8.07)	Z = −0.308	0.758
Neutrophil count (×10^9^/L)	4.19 (3.08, 11.73)	4.47 (3.11, 5.71)	Z = −0.440	0.66
Lymphocyte count (×10^9^/L)	1.07 ± 0.31	1.33 ± 0.46	*t* = −1.376	0.18
Red blood cell count (×10¹²/L)	4.53 (4.19, 4.72)	4.28 (3.73, 4.52)	Z = −1.364	0.173
Red cell distribution width (%)	14.00 (13.38, 18.93)	13.50 (13.23, 15.45)	Z = −1.038	0.299
Mean corpuscular volume (fL)	86.35 (64.59, 88.50)	89.90 (88.10, 92.80)	Z = −2.464	0.014
Hemoglobin (g/L)	125 (102.75, 134.75)	127 (107.25, 136.75)	Z = −0.330	0.741
Platelet count (×10^9^/L)	261.5 (142.25, 297.75)	222.5 (204.25, 295.50)	Z = 0.000	1
Procalcitonin (ng/mL)	0.09 (0.03, 0.36)	0.03 (0.02, 0.06)	Z = −1.174	0.24
Interleukin-6 (pg/mL)	21.97 (9.44, 31.60)	9.60 (6.01, 20.32)	Z = −1.804	0.071
C-reactive protein (mg/L)	48.76 (8.32, 117.74)	8.36 (2.30, 41.27)	Z = −1.518	0.129

There were no statistically significant differences in total white blood cell count, neutrophil count, lymphocyte count, red blood cell count, hemoglobin concentration, platelet count, procalcitonin levels, interleukin-6 levels, or C-reactive protein concentrations between patients with and without CDI (all P > 0.05). Although inflammatory markers, including interleukin-6 and C-reactive protein, tended to be higher in the CDI group, these differences did not reach statistical significance.

In contrast, mean corpuscular volume (MCV) was significantly lower in the CDI group compared with the non-CDI group (86.35 [64.59, 88.50] fL vs. 89.90 [88.10, 92.80] fL, P = 0.014). No significant differences were observed in red blood cell distribution width between the two groups.

### Liver and renal function parameters

Comparisons of liver and renal function parameters between the CDI and non-CDI groups are presented in **Table 5**. Overall, most biochemical indices reflecting hepatic and renal function were comparable between the two groups.

**Table 5:** Comparison of liver and renal function parameters between CDI and non-CDI groups.

Parameters	CDI (n = 10)	Non-CDI (n = 20)	Statistic	*P* value
Globulin (g/L)	26.64 ± 4.27	29.39 ± 6.12	*t* = −1.496	0.146
Albumin (g/L)	36.20 (29.40, 37.73)	38.10 (36.13, 43.15)	Z = −2.046	0.041
Blood urea nitrogen (mmol/L)	3.93 (2.61, 5.93)	5.60 (4.73, 6.09)	Z = −1.936	0.053
Serum creatinine (μmol/L)	54.50 (51.75, 70.00)	62.50 (46.50, 76.00)	Z = −0.506	0.613
Total bilirubin (μmol/L)	12.26 ± 4.30	12.74 ± 4.19	*t* = −0.654	0.518
Unconjugated bilirubin (μmol/L)	8.07 ± 3.53	7.54 ± 3.16	*t* = 0.294	0.771
Conjugated bilirubin (μmol/L)	5.45 (4.03, 7.78)	3.90 (2.64, 6.50)	Z = −1.167	0.243
Alanine aminotransferase (U/L)	17.30 (10.23, 33.75)	19.30 (12.33, 28.25)	Z = −0.264	0.792
Aspartate aminotransferase (U/L)	23.80 (20.88, 43.00)	23.42 (18.13, 31.15)	Z = −0.726	0.468

Serum albumin levels were significantly lower in the CDI group compared with the non-CDI group (36.20 [29.40, 37.73] g/L vs. 38.10 [36.13, 43.15] g/L, P = 0.041). In contrast, no statistically significant differences were observed between the two groups with respect to globulin levels, blood urea nitrogen, serum creatinine, total bilirubin, unconjugated bilirubin, conjugated bilirubin, alanine aminotransferase, or aspartate aminotransferase (all P > 0.05).

Overall hepatic and renal indices were largely comparable between groups. The lower albumin value in the CDI group was an unadjusted group difference and may reflect age, tuberculosis severity, inflammation, comorbidity, or malnutrition; it should not be interpreted as an independent CDI susceptibility factor.

### Laboratory parameters and PPI exposure

C-reactive protein was elevated in 8 of 10 CDI patients, with 5 patients showing levels above 50 mg/L. Hypoalbuminemia was documented in 8 patients, and 4 had white blood cell counts above 10 × 10^9^/L. Because all patients had active pulmonary tuberculosis and some had concomitant infectious or inflammatory conditions, these abnormalities were not considered specific to CDI. Consistent with this, CRP did not differ significantly between the CDI and non-CDI groups (P = 0.129).

Nine of the ten CDI patients had received omeprazole or another PPI continuously for at least two weeks during anti-tuberculosis treatment. Six of these patients had overt gastrointestinal symptoms. PPI exposure was therefore common in the CDI group, although its independent relationship with CDI could not be determined in this retrospective cohort.

### Factors associated with clostridioides difficile infection

Variables with P < 0.05 in univariate analyses, including body mass index (BMI), mean corpuscular volume (MCV), and serum albumin level, were entered into a multivariable binary logistic regression model to explore factors potentially associated with Clostridioides difficile infection. The presence of CDI was defined as the dependent variable (CDI = 1, non-CDI = 0). The results of the multivariable logistic regression analysis are summarized in **Table 6**.

**Table 6:** Multivariable logistic regression analysis for factors associated with CDI.

Variable	B	SE	Wald	df	*P* value	OR (Exp[B])	95% CI (Lower–Upper)
BMI	−0.276	0.191	2.1	1	0.147	0.759	0.522–1.102
Mean corpuscular volume (fL)	−0.051	0.049	1.088	1	0.297	0.95	0.862–1.046
Albumin (g/L)	−0.065	0.102	0.401	1	0.527	0.937	0.767–1.145
Constant	11.991	5.252	5.212	1	0.022	161,286.99	

In the multivariable analysis, none of the included variables remained independently associated with CDI. BMI (OR = 0.759, 95% CI: 0.522–1.102, P = 0.147), MCV (OR = 0.950, 95% CI: 0.862–1.046, P = 0.297), and serum albumin (OR = 0.937, 95% CI: 0.767–1.145, P = 0.527) were not statistically significant in the adjusted model (**Table 6**). The overall explanatory power of the model was modest (pseudo-R² = 0.337).

Collinearity diagnostics demonstrated that all variables included in the regression model had tolerance values greater than 0.1 and variance inflation factor (VIF) values below 1.5, indicating no significant multicollinearity among the independent variables. Detailed collinearity statistics are presented in **Table 7**.

**Table 7:** Collinearity diagnostics of variables included in the logistic regression model.

Variable	B	SE	Standardized β	*t*	*P* value	Tolerance	VIF
Constant	2.235	0.524	—	4.261	<0.001	—	—
BMI	−0.041	0.022	−0.335	−1.875	0.072	0.786	1.273
Mean corpuscular volume (fL)	−0.007	0.004	−0.310	−1.877	0.072	0.921	1.086
Albumin (g/L)	−0.010	0.011	−0.151	−0.849	0.404	0.791	1.264

### Treatment and outcomes

All patients received anti-tuberculosis treatment according to drug-susceptibility results, and none had rifampin-resistant tuberculosis. Most received HRZE or modified four-drug regimens containing isoniazid, rifampin, pyrazinamide, and ethambutolamid.

Following CDI diagnosis, management included discontinuation of PPI therapy in six patients, correction of fluid and electrolyte disturbances, probiotic supplementation, and antimicrobial de-escalation or modification in selected cases. Non-tuberculous systemic antimicrobial therapy was also documented in some CDI patients for concomitant extraintestinal infections. None of the CDI patients received oral vancomycin or fidaxomicin.

During hospitalization, seven CDI patients improved and were discharged, two were transferred to higher-level hospitals because of persistent symptoms and substantial comorbidities, and one died. The deceased patient was a 74-year-old man with severe pulmonary tuberculosis and multiple comorbidities, including chronic obstructive pulmonary disease, heart failure, intracerebral hemorrhage, and fungal pneumonia. Despite broad-spectrum antimicrobial and antifungal therapy, he developed persistent diarrhea, progressive respiratory failure, and multi-organ dysfunction and died on hospital day 33.

## Discussion

In this retrospective comparative study, ten patients with clinically classified, PCR-positive CDI and twenty contemporaneous non-CDI controls were evaluated during anti-tuberculosis treatment. The design permits description of group differences but cannot establish that rifampin, PPIs, hospitalization, or prior non-tuberculous antibiotics caused CDI. Reports involving TB patients remain limited. Mechanistic and observational studies suggest that prolonged antimicrobial exposure may alter mucosal integrity and microbial composition, particularly when fluoroquinolones or rifamycin derivatives are used with gastric acid suppressants such as PPIs (Kim et al., 1981; Kim et al., 2021). Recent studies have also emphasized the persistent difficulty in distinguishing true infection from colonization in high-risk hospital settings, including TB wards, due to overlapping symptoms and delayed testing strategies (Chin et al., 2023; Miles-Jay et al., 2023). Our observations support diagnostic vigilance in symptomatic TB inpatients but should be considered hypothesis-generating because exposure timing was incomplete and major confounders were not controlled.

In this cohort, gastrointestinal symptoms documented during anti-TB therapy could have been attributed clinically to drug intolerance, malnutrition, tuberculosis, or enteric infection. Stool PCR detected C. difficile toxin genes, but incomplete patient-level exposure timing and the absence of toxin testing limited distinction between causation, association, and possible colonization. Rifampin and fluoroquinolones can disrupt anaerobic commensals and suppress gut microbial diversity, creating conditions for C. difficile overgrowth (Vincent et al., 2016). These changes can persist well beyond the course of antibiotics, especially in patients with compromised mucosal immunity (Seekatz et al., 2015). The CDI group had lower BMI, MCV, and serum albumin in unadjusted comparisons. These differences may instead reflect age, tuberculosis severity, chronic inflammation, comorbidity, or malnutrition, and none remained independently associated with CDI in multivariable analysis. They should therefore not be interpreted as independent susceptibility factors. Symptom-triggered CDI testing remains reasonable in high-risk TB inpatients.

Structural abnormalities of the lung, such as cavitary lesions and bronchiectasis, were common in this cohort. Six patients showed radiologic features consistent with bronchial distortion, while three had cavitary changes. These findings, though typical of advanced TB, often prompt the use of prolonged antimicrobial therapy and gastric acid suppression to manage recurrent pulmonary infections and prevent aspiration. However, both practices are known to disturb the intestinal microbiota and increase CDI risk (Kaya et al., 2021; Miles-Jay et al., 2023). One case report described CDI emerging in a hidradenitis suppurativa patient receiving rifampin and clindamycin, highlighting the risk of CDI in settings of non-gastrointestinal disease treated with anti-TB agents (Bessaleli and Scheinfeld, 2018). In addition, atypical systemic manifestations such as reactive arthritis and appendiceal pain have been documented in CDI patients, often leading to diagnostic confusion (Legendre et al., 2016; Pham and Hua, 2021). In TB wards, gastrointestinal complaints are easily masked by the dominant respiratory focus, and clinical suspicion for CDI may remain low unless diarrhea is severe. Our findings suggest that delayed recognition may be further compounded by clinicians’ reliance on respiratory disease severity rather than concurrent metabolic or gastrointestinal signals, underscoring the need for broader diagnostic vigilance.

In clinical settings, timely recognition of CDI remains a challenge, especially in patients undergoing anti-TB treatment. Diarrhea is frequently regarded as a side effect of antituberculous drugs or malnutrition, leading to underrecognition of enteric infections. In our cohort, several patients experienced gastrointestinal symptoms for days before stool PCR testing was performed. This delay may be explained by diagnostic anchoring, particularly when systemic inflammation or abdominal discomfort is attributed to preexisting disease. A report by Pham et al. described a case of CDI misdiagnosed as appendicitis in an immunocompromised host, highlighting how nonspecific symptoms can mislead clinical judgment and postpone appropriate intervention (Pham and Hua, 2021). Similarly, the case published by Bessaleli et al. demonstrated CDI development in a patient treated with rifampin and clindamycin, two agents commonly used in TB and related infections (Bessaleli and Scheinfeld, 2018). Diagnostic strategies have also been shown to influence recognition rates; patients tested only with toxin immunoassays may be falsely negative despite clinical signs, delaying treatment (Planche et al., 2013). In addition, cumulative exposure to multiple classes of antibiotics has been linked to a higher risk of hospital-acquired CDI, particularly in elderly populations with comorbidities (Slimings and Riley, 2014). In our study, symptoms improved in several patients after PPI discontinuation and supportive management; however, treatment was not standardized and the improvement cannot be attributed causally to PPI withdrawal. The observation supports timely diagnostic evaluation rather than a treatment-effect conclusion.

This study has several limitations. First, its retrospective, single-center design and small sample limit generalizability and statistical power. Controls were contemporaneous but not individually matched for age, sex, or length of stay. At the prespecified eligibility review, records with a clearly established alternative cause of the diarrheal or suspected gastrointestinal infectious presentation were excluded before group assignment; this reduced, but could not eliminate, etiologic misclassification. Extraintestinal infections were retained because they may coexist with CDI and could confound systemic inflammatory markers and antimicrobial exposure. Second, exact patient-level timing of symptom onset relative to admission, rifampin, PPI initiation, and non-tuberculous antibacterials was not consistently retrievable. Antibiotic class, indication, dose, duration, and start-stop dates were variably documented. We coded missing details as unavailable and performed neither imputation nor class-, dose-, or duration-specific analyses; residual confounding remains likely. Third, PCR does not distinguish colonization from active toxin-mediated disease, stool-frequency documentation was incomplete, and toxin testing or toxigenic culture was unavailable. Fourth, follow-up ended at discharge, transfer, or in-hospital death, so recurrence and longer-term outcomes were not assessed. Finally, lower BMI, MCV, and albumin were unadjusted group differences and no independent predictors were identified in multivariable analysis. Accordingly, the data do not establish that nutritional status, rifampin, PPIs, hospitalization, or non-tuberculous antibacterial exposure caused or independently increased CDI risk. Multicenter prospective studies with standardized exposure timelines, toxin-based diagnostic algorithms, and post-discharge follow-up are needed.

## Data Availability

The original contributions presented in the study are included in the article/supplementary material. Further inquiries can be directed to the corresponding author.
